# The future role of the GP Quality and Outcomes Framework in England

**DOI:** 10.3399/BJGPO.2023.0054

**Published:** 2023-07-12

**Authors:** Azeem Majeed, Mariam Molokhia

**Affiliations:** 1 Professor of Primary Care and Public Health, Department of Primary Care, Public Health, Imperial College London, London, UK; 2 Clinical Reader in Epidemiology & Primary Care, School of Life Course & Population Sciences, King’s College, London, UK

**Keywords:** Primary healthcare, General practitioners, Quality and Outcomes Framework, Outcomes, Quality

The Quality and Outcomes Framework (QOF) was introduced in 2004 as part of a new NHS GP contract. The aim of the QOF was to financially reward general practices for the delivery of evidence-based standards of care. While pay for performance is well-established in the USA and some other countries, the scope, ambition, and cost of the QOF in the UK was unique internationally when it was launched.^1^ But with the NHS now struggling in many key areas, the future of the QOF in England is uncertain, with calls from many GPs to radically cut it back or even abolish it.^2,3^

Initially, up to one-quarter of general practice income was dependent on performance in the QOF. More recently, however, the QOF has been caught up in the wider problems affecting NHS primary care in England, such as the increased volume and complexity of workload, and workforce shortages. Furthermore, with health now a devolved function, the role of QOF in the different UK countries has changed; for example, Scotland abolished its own primary care QOF programme in 2016. The proportion of funding derived from QOF for general practices in England has also declined. What future role should the QOF play, if any, in England’s NHS?

A key role for any primary care system is providing comprehensive health services that cover both acute and long-term conditions. This means a focus not just on urgent care and immediate patient needs, but also on the prevention, early diagnosis, and management of the long-term medical problems such as cardiovascular disease, diabetes, and chronic lung diseases that are major causes of ill health, multimorbidity, reduced quality of life, NHS workload, and death.^4^

Since the start of the COVID-19 pandemic in 2020, we have seen the NHS focusing much more on urgent care. While this was necessary when options for managing COVID-19 such as vaccination were unavailable, the NHS should now once again be looking to provide high quality care for long-term conditions. The pandemic period saw a decline in the number of patients starting treatment for conditions such as high blood pressure.^5^ Moreover, over the last year, death rates in England have been higher than expected, partly due to increased deaths from conditions such as cardiovascular disease.^6^ These public health challenges reinforce the importance of the QOF, particularly the areas in the QOF focused on secondary prevention and the management of long-term conditions.

Recent evidence, for example, shows that patients who achieve QOF targets for the management of type 2 diabetes have lower rates of death, emergency hospital admission, retinopathy, and amputation.^7–10^ These findings illustrate that the QOF can be a very powerful secondary prevention tool, leading to better health outcomes for patients and lower pressures on other parts of the NHS. Furthermore, the QOF also provides data for the measurement of the quality of healthcare, which is essential for planning health services, addressing health inequalities, and ensuring value for money for public investment in NHS primary care. The structured data entry in medical records required for QOF also facilitates the use of the data for clinical research, the importance of which was shown during the COVID-19 pandemic.^11,12^

Abolishing or cutting back QOF would therefore be a decision with significant negative consequences. We need to support primary care teams so that they can provide the structured care promoted by QOF as well as dealing with any urgent and immediate problems that patients present with. This requires adequate funding for primary care teams, including reviewing how funding is allocated; for example, a shift to a greater proportion of funding based on activity and quality of care.^13^ Workforce issues also need to be addressed by promoting retention of primary care staff and expanding recruitment into new primary care roles. This includes, for example, better integration of pharmacy and general practice services. Finally, we need to make effective use of information technology and the wider primary care team. Developments in NHS information technology means that it should be possible to deliver elements of QOF 'at scale'. This could include, for example, centralised recalls and reminders for patients; and risk stratification so that patients who will benefit most from QOF are targeted first for secondary prevention. This strategy has been successfully used for prioritising COVID-19 vaccinations.^14^

The QOF is often criticised as being restrictive in its reporting domains, lacking the ability to evaluate important dimensions of quality of care, and its future is uncertain. However, when it was launched, it received wide acclaim and was seen as an area where the NHS was world-leading. Furthermore, recent research from Scotland shows that the abolition of financial incentives can lead to reductions in recorded quality of care for many performance indicators.^15^ We need to retain the best elements of QOF, particularly areas focused on the early detection and management of long-term conditions, with delivery of the QOF better supported by information technology and the wider primary care team. An effective QOF programme remains essential for the NHS if we are to reduce health inequalities and improve health outcomes in England.

## References

[1] Roland M (2004). Linking physicians’ pay to the quality of care—a major experiment in the United Kingdom. N Engl J Med.

[2] Tilley C QOF should be scrapped but metrics retained, says Jeremy Hunt. Pulse [online] 26 Apr 2022. https://www.pulsetoday.co.uk/news/politics/qof-should-be-scrapped-but-metrics-retained-says-jeremy-hunt/.

[3] Pulse 2023: The year the BMA gets radical? Pulse [online] 4 Jan 2023. https://www.pulsetoday.co.uk/analysis/cover-feature/2023-the-year-the-bma-gets-radical/.

[4] Whitty CJM, Smith G, McBride M, Atherton F (2023). Restoring and extending secondary prevention. BMJ.

[5] Dale CE, Takhar R, Carragher R, Katsoulis M (2023). The impact of the COVID-19Pandemic on cardiovascular disease prevention and management. Nat Med.

[6] Raleigh V (2022). What is driving excess deaths in England and Wales?. BMJ.

[7] McKay AJ, Gunn LH, Vamos EP, Valabhji J (2021). Associations between attainment of incentivised primary care diabetes indicators and mortality in an English cohort. Diabetes Res Clin Pract.

[8] Gunn LH, McKay AJ, Molokhia M, Valabhji J (2021). Associations between attainment of Incentivised primary care indicators and emergency hospital admissions among type 2 diabetes patients: a population-based historical cohort study. J R Soc Med.

[9] Gunn LH, Vamos EP, Majeed A, Normahani P (2021). Associations between attainment of Incentivized primary care indicators and incident lower limb amputation among those with type 2 diabetes: a population-based historical cohort study. BMJ Open Diabetes Res Care.

[10] McKay AJ, Gunn LH, Sathish T, Vamos E (2021). Associations between attainment of Incentivised primary care indicators and incident diabetic retinopathy in England: a population-based historical cohort study. BMC Med.

[11] Williamson EJ, Walker AJ, Bhaskaran K, Bacon S (2020). Factors associated with COVID-19-related death using Opensafely. Nature.

[12] Clift AK, Coupland CAC, Keogh RH, Diaz-Ordaz K (2020). Living risk prediction algorithm (QCOVID) for risk of hospital admission and mortality from Coronavirus 19 in adults: national derivation and validation cohort study. BMJ.

[13] Razai MS, Majeed A (2022). General practice in England: the current crisis, opportunities, and challenges. J Ambul Care Manage.

[14] Majeed A, Pollock K, Hodes S, Papaluca M (2022). Implementation of COVID-19 vaccination in the United Kingdom. BMJ.

[15] Morales DR, Minchin M, Kontopantelis E, Roland M (2023). Estimated impact from the withdrawal of primary care financial incentives on selected indicators of quality of care in Scotland: controlled interrupted time series analysis. BMJ.

